# Mechanisms of multiyear variations of Northern Australia wet-season rainfall

**DOI:** 10.1038/s41598-020-61482-5

**Published:** 2020-03-20

**Authors:** S. Sharmila, Harry H. Hendon

**Affiliations:** 1 Centre for Applied Climate Sciences, University of Southern Queensland, Toowoomba, Australia; 2 Bureau of Meteorology, Melbourne, Australia

**Keywords:** Atmospheric dynamics, Climate sciences, Atmospheric science

## Abstract

Northern Australia wet season (November–April) rainfall exhibits strong variability on multiyear timescales. In order to reveal the underlying mechanisms of this variability, we investigate observational records for the period 1900–2017. At multiyear timescales, the rainfall varies coherently across north-western Australia (NW) and north-eastern Australia (NE), but the variability in these two regions is largely independent. The variability in the NE appears to be primarily controlled by the remote influence of low frequency variations of El Niño-Southern Oscillation (ENSO). In contrast, multiyear variations in the NW appear to be largely driven locally and stem from a combination of rainfall-wind-evaporation feedback, whereby enhanced land-based rainfall is associated with westerly wind anomalies to the west that enhance local evaporation over the ocean to feed the enhanced land based rainfall, and soil moisture-rainfall feedback. Soil-moisture and associated evapotranspiration over northern Australia appear to act as sources of memory for sustaining multiyear wet and dry conditions in the NW. Our results imply that predictability of multiyear rainfall variations over the NW may derive from the initial soil moisture state and its memory, while predictability in the NE will be limited by the predictability of the low frequency variations of ENSO.

## Introduction

**Figure 1:**
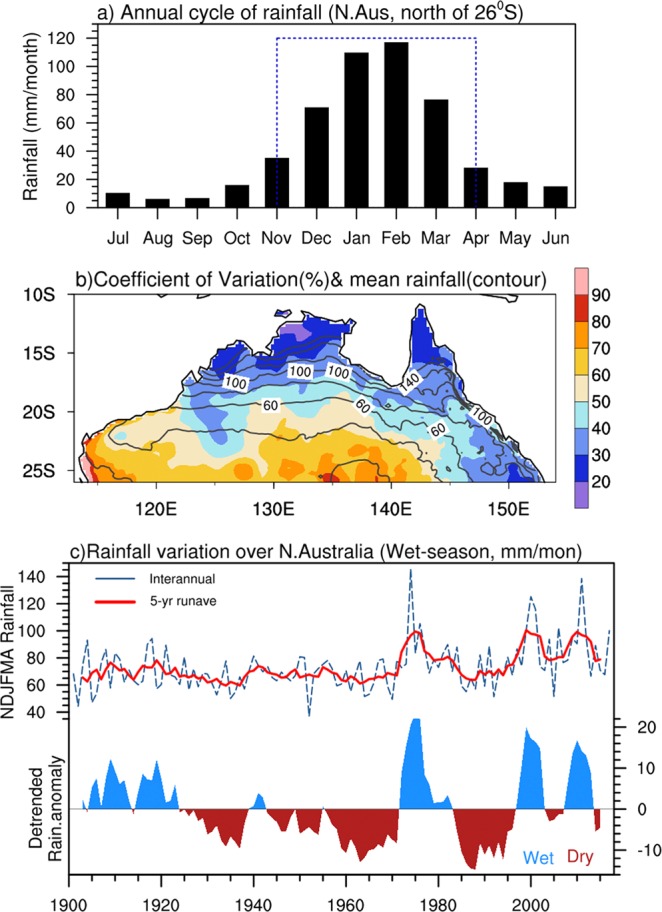
(**a**) Annual cycle of monthly mean rainfall (mm month^−1^) over Northern Australia (land points north of 26°S) for 1900–2017. The wet season is highlighted with dashed blue box. (**b**) spatial distribution of coefficient of variability (in %, shaded) and mean rainfall (contour, mm month^−1^) during the wet-season. (**c**) Timeseries of wet season rainfall averaged over northern Australia (dashed blue line) and smoothed with a 5-year running mean (red line). The bottom panel has been detrended, with wet (blue) and dry (red) epochs indicated. All figures are based on AWAP rainfall analyses for the 1901–2017 period.

Northern Australia, defined here as the tropical portion of Australia north of 26 °S (following Bureau of Meteorology convention), receives ~80% of its annual mean rainfall during the extended summer monsoon season November–April (Fig. 1a). This extended season is commonly referred as the northern wet season. The wet season last longer than the summer monsoon, because an appreciable portion of the wet season rainfall occurs prior to monsoon onset and after monsoon withdrawal, defined when the low-level circulation reverses from trade easterlies to monsoon westerlies and back again across the most northern portion of the continent^1^. Wet season rainfall is highly reliable across the most northerly portions of the continent^2^ (i.e., north of 17 °S), with a coefficient of variation (standard deviation divided by the mean) about ~20% (Fig. 1b). However, farther to the south and inland, the rainfall is highly variable with the coefficient of variation approaching ~50%. This variable rainfall has been attributed to impacts from the naturally occurring dominant mode of climate variability over the tropical Pacific - El Niño Southern Oscillation (ENSO)^2^, which strongly affects interannual variations of rainfall^2^. However, northern Australia rainfall also varies strongly on multiyear and decadal timescales (e.g., Fig. 1c, top panel), especially associated with low frequency variations of ENSO and the Interdecadal Pacific Oscillation (IPO)^3^.

The focus of this study is the wet-season multiyear rainfall variability, highlighted in Fig. 1c by applying a 5-year running mean (red curve) on wet-season mean rainfall time series (dashed blue). Multiyear wet and dry epochs are readily discernible, but there is also a noticeable upward trend that has been well documented (http://www.bom.gov.au/climate/current/annual/aus/) and is not the focus of the present study. Although there is also an apparent increase in low-frequency variability in the recent decades, after removal of the long-term trend (Fig. 1c, bottom panel) there is much less impression of enhanced rainfall variability in recent decades. Prominent multiyear variability is evident throughout the record. e.g., sustained dry conditions during the 1930’s to 1960’s and the late 1980’s to late 1990’s, which were punctuated by strong wet epochs in the 1970’s, the 2000’s, and the 2010’s. These wet and dry epochs are largely due to successive years of dry or wet conditions, rather than isolated extreme years. These multiyear rainfall variations can have profound impacts on agricultural production and associated business revenues in northern Australia^4^. The motivation for this study is to advance knowledge of the causes of this multiyear variability so to better inform and prepare key agricultural sectors to better manage the impacts, for instance, on the vast grazing industry across northern Australia.

Previous studies have highlighted the primary role of ENSO for driving interannual variations of Australian rainfall, with dry conditions tending to prevail during El Niño and wet conditions during La Niña^5^. However, Hendon *et al*.^6,7^ argue that the remote impacts of ENSO on northern Australia rainfall weaken going from late spring into summer, especially in the western portion of northern Australia, as a result of local air sea interaction to the north-west of Australia: remote forcing from El Niño (La Niña) drives easterly (westerly) surface wind anomalies to the north-west of Australia, which act to warm (cool) the local ocean after onset of Australian summer monsoon, thus countering the remotely-forced drying (wetting) induced by central Pacific sea surface temperature (SST) anomalies.

A recent study^8^ suggests an additional mechanism for interannual modulation of Australian summer rainfall that is independent of ENSO: enhanced land-based monsoon rainfall is promoted by a feedback from enhanced evaporation over the warm seas to the north west of Australia under the influence of enhanced surface westerlies that feed the enhanced land-based monsoon rainfall. This mechanism for inducing summer season rainfall variability, which operates only during the monsoon season when the mean surface winds are westerly, is independent of remote forcing from ENSO and is possibly more important to western portions of the continent, where the enhanced evaporation over the ocean to the west can more directly feed the land-based rainfall. Land-surface processes can also impact the local atmospheric variability by altering surface energy and water fluxes^9^. For example, the fluctuations of soil moisture can contribute to an increase in the persistence of surface temperature and precipitation^10–12^. Deep layer soil moisture is a promising source of rainfall variability and predictability at longer timescales because it integrates cumulative hydrological effects and so potentially can provide long term memory, but its impacts are difficult to quantify. Although these mechanisms for driving interannual variations of Australian summer rainfall are well established, the extent to which these processes impact multi-year timescales are still unknown.

Building on these previous studies, the present work aims to identify and understand the mechanisms of multiyear wet season rainfall across northern Australia, with the goal of providing insight for the potential to predict it. We specifically explore the spatial characteristics of the multiyear rainfall variations across northern Australia, considering the previously mentioned studies^13^ that suggest mechanisms that can selectively affect the western or eastern portions of northern Australia. An improved knowledge of the driving mechanisms of multiyear wet season rainfall variations will translate into better assessment of the climate risk management related to prolonged periods of severe rainfall variability over northern Australia.

## Results and Discussions

### Dominant modes of multiyear rainfall - regional contrast

We begin by objectively identifying the leading patterns of spatially coherent, multiyear rainfall variability by applying rotated empirical orthogonal function (rEOF) analysis using the varimax method^14^ to the low-pass filtered AWAP-observational gridded rainfall analyses^15^ (see Methods) covering Australia. The rEOF analysis is an objective technique that can effectively identify leading patterns of localized variability. We use monthly AWAP rainfall data on a 0.25° × 0.25° covering the period 1900–2017. We focus on wet-season mean (Nov–Apr) rainfall (Fig. 1a). Prior to the rEOF analysis, we linearly detrend and apply a 5-year running mean to the Nov–Apr averaged rainfall in order to remove the interannual variations associated with individual ENSO events and any long-term trend so to better focus on multiyear variations.

**Figure 2:**
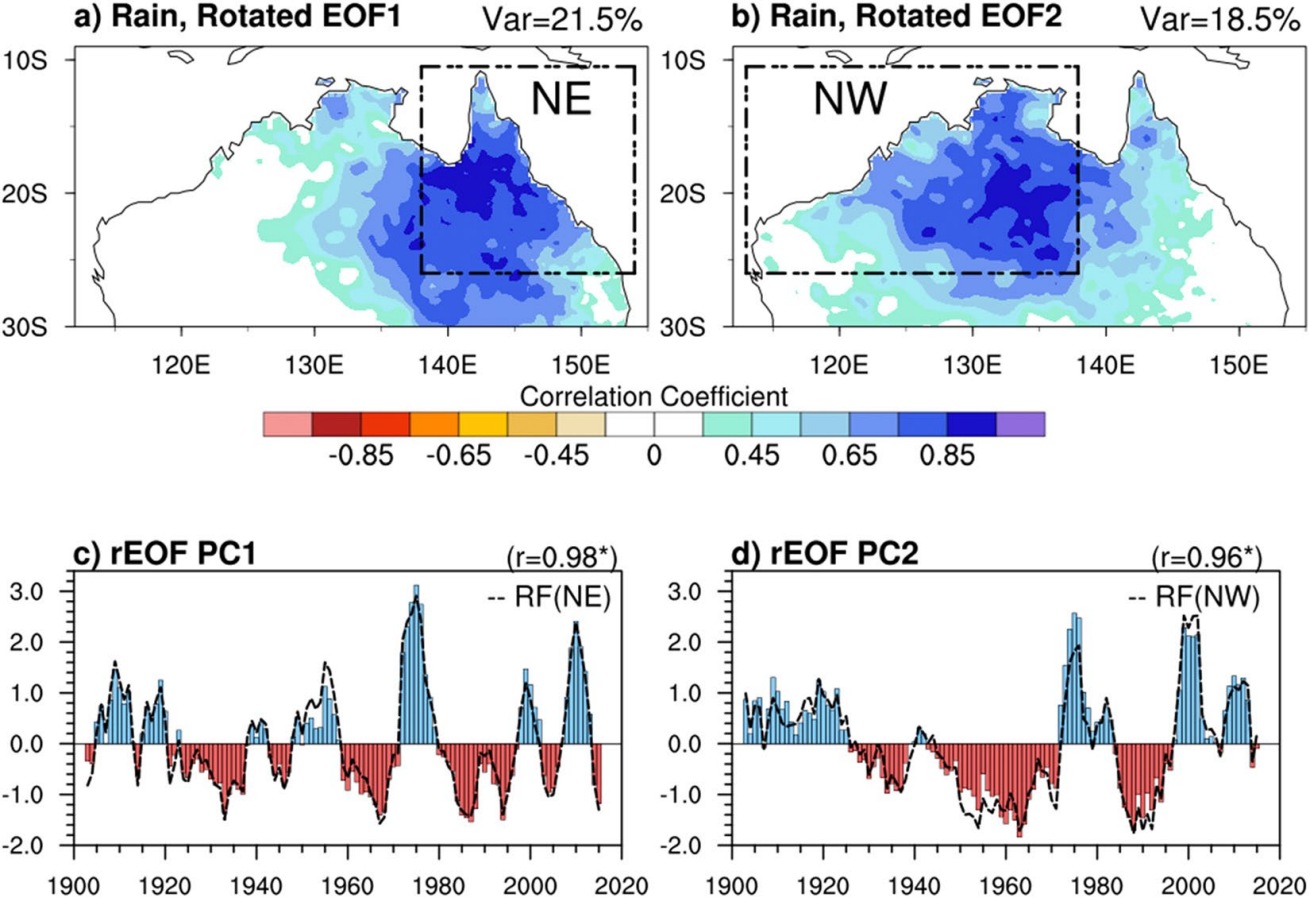
(**a**,**b**) Spatial patterns and (**c**,**d**) associated timeseries of normalized leading principal component of the first two rotated EOFs of the low-pass filtered wet season mean rainfall anomalies for the 1901–2017 period. The explained variances are indicated in the upper right. The defined study regions NE (land points east of 138°E) and NW (land points west of 138°E) are indicated in (**a**,**b**) as boxes. The box averaged standardized rainfall indices (black dash curve) for NE, and NW with their correlation coefficients are also overlaid in (**c**,**d**) respectively.

The leading two spatial loading patterns (displayed here by correlation of AWAP rainfall onto the PC timeseries) and their principal component times series are displayed in Fig. 2. The rEOF1 (Fig. 2a), which explains 21.5% of total rainfall variance, has the largest loading over the eastern half of the continent and weak loading in the west. The rEOF2 (Fig. 2b), which accounts for 18.5% of the total rainfall variance, has strongest loadings in the central and western portions of the continent and is generally more spatially extensive than is rEOF1. Although the two PC time series share some common behaviour (e.g. the wet episodes in the 1970s, 1990s, and 2010’s), the PC2 exhibits markedly lower frequency variations than rotated PC1 (Fig. 2c,d). Computation of the coherence squared between PC1 and PC2 (figure not shown) reveals significant coherence at interannual time scales (i.e. at periods less than ~3 years) but coherence is weaker and not generally significant at multiyear periods. This indicates that a significant portion of the interannual variation of northern Australian rainfall varies coherently across the continent^13^ but that at lower multiyear frequencies fundamentally different processes are driving NW rainfall compared to NE rainfall.

In order to simplify our analysis, we can represent rEOF1 by simply area averaging the rainfall anomalies north of 26°S for land points to the east of 138°E (referred to as NE, shown as box in Fig. 2a) and can represent rEOF2 by similarly averaging to the west of 138 °E (referred to as NW, shown as box in Fig. 2b). The timeseries of standardised rainfall indices (black dashed curve, Fig. 2c,d) based on the NE and NW domain averaged rainfall time series (Supplementary Fig. S1), look nearly identical to the timeseries of rotated PC1 and PC2 (bar, Fig. 2c,d). The respective correlations both exceed 0.96 (significant p < 5%) indicating the usefulness of rEOFs in identifying localized modes of variability. The coherence spectra of area-averaged northern Australia rainfall with rainfall in NE and in NW (Supplementary Fig. S2) confirms that NW rainfall is systematically more coherent with the area-averaged northern Australia rainfall across all frequencies compared to NE rainfall, with the difference becoming greater at the higher interannnual frequencies.

### Multiyear rainfall variability and SST teleconnection

**Figure 3:**
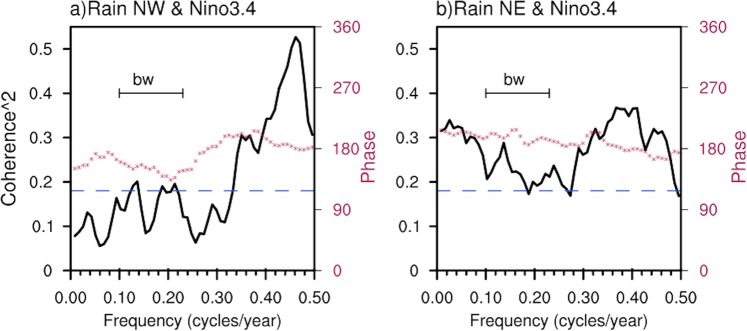
Coherence-squared (black curve) and phase (red dotted) spectra of Niño3.4 index with area averaged rainfall in (**a**) NW, and (**b**) NE. The scale for coherence squared is on the left ordinate and for the phase (degrees, below 180^°^ means Niño3.4 is leading and above 180° means Niño3.4 is lagging) is on the right ordinate respectively. The dashed blue line indicates the 95% confidence level for the coherence squared estimates. The bandwidth of the spectra is also shown at the top left.

The relationship of multiyear rainfall variability with the SST variations in the tropical eastern Pacific associated with low-frequency ENSO or the IPO^3^ is quantified by computing the coherence squared spectrum of NW and NE rainfall with the Niño3.4 SST index (Fig. 3a,b; comparable results are obtained using the IPO index^16^ (not shown). Rainfall in both NE and NW is out of phase with Niño3.4 SST variations (i.e. dry during El Niño), but rainfall in the NW is only coherent with Niño3.4 SST variations at interannual periods (periods less than ~3 years). That is, multiyear (periods longer than 3 years) variations of NW rainfall are largely independent of SST variations in the tropical eastern Pacific. In contrast, rainfall in the NE is significantly coherent with Niño3.4 SST variations across all frequencies.

**Figure 4:**
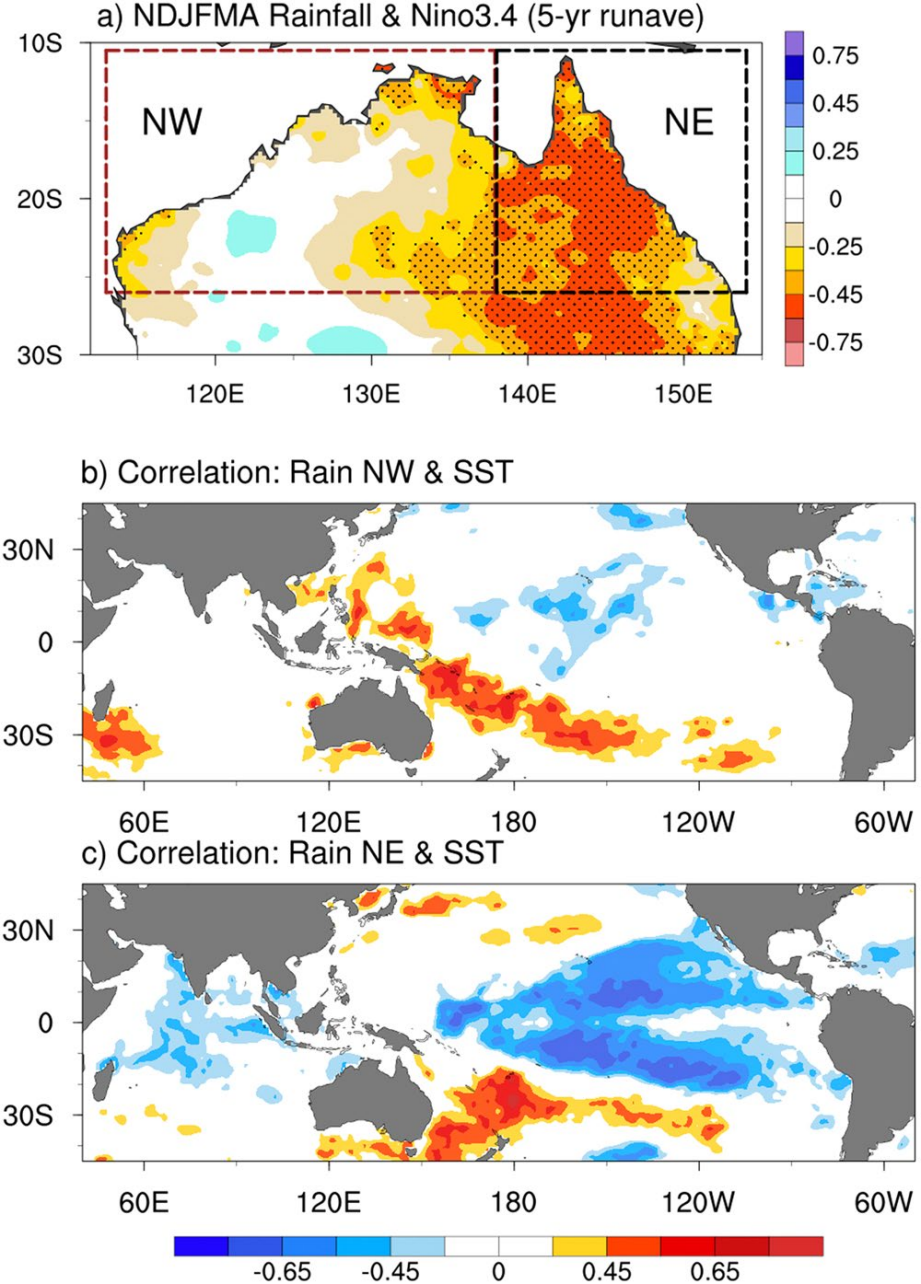
(**a**) Spatial correlation between multiyear wet-season Australian rainfall anomalies and low-frequency Niño3.4 index where values significant above 95% confidence level are stippled. Spatial correlation between low-pass filtered global SST and (**b**) NW rainfall, and (**c**) NE rainfall, where only significant values are plotted. Data are linearly detrended and low-pass filtered with a 5-year running mean for the period 1901–2017.

We confirm this lack of coherence of multiyear NW rainfall variability with Niño3.4 SST variations by computing the correlation of gridded rainfall with the low pass filtered (5-year running mean) Niño3.4 SST index (Fig. 4a). Strong negative correlation is confined to the eastern half of northern Australia and the correlation is near zero in the NW region. This correlation pattern with the low-frequency Niño3.4 index is similar to the rainfall composite for the warm minus cold phase of IPO (Supplementary Fig. S3), confirming that low-frequency filtered Niño3.4 index is a good surrogate for the IPO. We further confirm the distinctive relationship of NE and NW multiyear rainfall with low-frequency tropical Pacific SST by computing the correlation of SST with NW rainfall (Fig. 4b) and with NE rainfall (Fig. 4c). Multiyear variations of NE rainfall are strongly correlated remotely with SST in the tropical eastern Pacific, with a pattern reminiscent of the cold phase of the IPO^3,17^. In contrast, NW rainfall shows little or no correlation with tropical eastern Pacific SST. NW rainfall does show positive correlation with SST in the south west tropical Pacific to the north-east of Australia, but this appears to be largely a response rather than a forcing of NW rainfall variation. Examination of the pattern of global rainfall associated with NW rainfall (Supplementary Fig. S4a) shows little anomaly in the South Pacific Convergence Zone (SPCZ), which normally lies above this region of positive SST correlation seen in Fig. 4b. In contrast, the rainfall pattern associated with enhanced NE rainfall does show an extension into the SPCZ (Supplementary Fig. S4b), consistent with forcing from the warm SSTs seen in Fig. 4c. Hence, NE rainfall is associated with a westward shift of the SPCZ that impinges on the eastern Australia and is a reminiscent of La Niña-like conditions as would be anticipated during the cold phase of the IPO.

### Multiyear wet - dry composites

**Figure 5:**
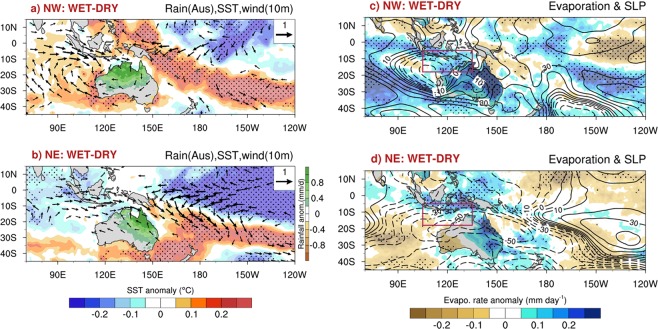
Composite of WET minus DRY conditions for the NW (top panels) and the NE (lower panels): for ((**a**,**b**) left column) Australian rainfall^15^ (mm d^−1^), SST (shaded, °C) and surface wind at 10-m (vectors, ms^−1^), and ((**c**,**d**) right column) evaporation (shaded, mm d^−1^) and mean sea level pressure (contoured, with contour interval of 5Pa). The scale for the wind vectors (ms^−1^) is in upper right. All data have been detrended and low pass filtered with a 5-year running mean for the period 1901–2010 available for ERA-20C^19^. Values significant above 95% confidence level are shown by stippling. Vectors are plotted only where significantly different than zero at 5% level.

In order to better understand the distinctive causes of the multiyear variations of rainfall in the NE and NW, we make composite of wet and dry conditions for the NE and NW. We select wet and dry years when the respective standardized indices of low-pass filtered (5-year running mean) rainfall indices are above +0.5 and below −0.5 respectively. We form composites of rainfall from AWAP analyses^15^, sea surface temperature from HadISST^18^, surface winds and evaporation from ERA-20C reanalyses^19^, and vertically integrated soil moisture and evapotranspiration over land from the AWRA-Lv6 analyses^20^ (See Methods). Because we find largely opposite anomalies between wet epochs and dry epochs with little asymmetry, we display the composited fields as wet minus dry (WET-DRY). For both the NW (Fig. 5a), and the NE (Fig. 5b) composites, wet conditions are associated with surface westerly wind anomalies that feed into the rainfall anomalies. However, for the NE, the westerly wind anomalies are much broader zonally, extending eastward from the central Indian Ocean to the central Pacific, and are accompanied by easterly wind anomalies in the tropical eastern Pacific in conjunction with cold SSTs there. Wet conditions in the NE thus are associated with an enhanced Walker circulation and a La Niña-like SST anomaly. In contrast, the westerly anomalies associated with NW wet conditions are confined locally to the NW of Australia and are part of a localized cyclonic circulation centred to the west of Australia over the subtropical Indian Ocean (Fig. 5c). The circulation pattern for wet conditions in the NW is consistent with the expected response^21^ to a localized rainfall anomaly over the NW of Australia. In contrast, the pattern of surface pressure and surface wind anomaly for wet conditions in the NE, spans the entire tropical Indo-Pacific and is reminiscent of the positive phase of the Southern Oscillation, with higher pressure in the equatorial eastern Pacific and lower pressure broadly over the equatorial western Pacific and eastern Indian Oceans^13^.

### Proposed mechanisms - remote vs local

What are the underlying processes that drive multiyear rainfall variations in the NW, which appear to be associated with local changes in circulation and the absence of any significant tropical SST anomalies, and in the NE, which appear to have a strong relationship with remote SST variability? One possible mechanism for promoting NW and NE rainfall that can be discarded is through an enhanced ocean-land surface temperature gradient, which is fundamental to monsoon circulation. However, during wet epochs for both NW and NE, the surface temperatures over land are reduced (Supplementary Fig. S5), presumably as a result of enhanced surface evaporation and/or reduced surface insolation due to increased cloud cover. So, we can exclude local variation of monsoon forcing as a possible mechanism.

On the other hand, previous studies have highlighted that internally generated atmospheric variability, in the absence of anomalous tropical SSTs, can produce prolonged wet and dry episodes^22,23^. A recent study^8^ finds that seasonally persistent anomalous convection over northern Australia during the austral summer monsoon, which is independent of ENSO, can be promoted locally through a rainfall-wind-evaporation feedback. We explore whether such a feedback might operate at multiyear timescales. We examine evaporation locally around and over Australia for the NW and NE wet-dry composites (Fig. 5c,d). For the NW case, enhanced evaporation (or latent heat flux) occurs locally upstream (to the west) over the ocean to the north west of Australia (Fig. 5c), which coincides with enhanced surface westerlies (Fig. 5a). By forming the composite for each month November–April (not shown), we confirm that this enhanced evaporation in association with enhanced surface westerlies primarily occurs during the summer monsoon portion of the wet season (i.e, during January–March), when the mean surface winds are monsoonal westerlies^8^.

Importantly, the local SST anomalies, underlying the enhanced surface westerlies to the north west of Australia associated with enhanced NW rainfall, are insignificant (Fig. 5a). In absence of significant local SST anomaly, the anomalous westerlies during the monsoon portion of the wet season act to increase surface evaporation because they act to increase the surface windspeed, while also transporting moisture eastward to support enhanced inland rainfall over NW. Thus, the mechanism of Sekizawa *et al*.^8^ appears to be operating on the multiyear timescale to sustain the multiyear rainfall variations in the NW. However, this local rainfall-wind-evaporation feedback does not appear to be prominent during NE wet years (Fig. 5d): enhanced NE rainfall is associated with more zonally extensive westerly anomalies that overlay colder than normal SST to the north west of Australia and the local evaporation anomalies are near zero. The westerly anomalies during NE wet periods across northern Australia appear to be part of the response to the La Niña-like cold SST anomaly in the central equatorial Pacific. The induced westerly anomalies to the north-west of Australia act to locally to cool the ocean there, thus mitigating any increase of evaporation due to enhanced windspeed, thereby weakening the impact of La Niña on rainfall in the NW^7,24^.

We quantify the relative contribution of local and remote forcing for promoting NW and NE rainfall variations using a simple multiple linear regression analysis for the period 1901–2010. Here, the NW and NE rainfall indices are the predictands, while the two predictors are the low-pass filtered Niño3.4 SST index (to capture the remote influence), and local evaporation to the north west of Australia (to capture the local influence; averaged over 5°–18°S, 105°–135°E; indicated by a box in Fig. 5c,d). The partial regression coefficients (b*) based on standardized predictors are shown in Table 1. The local evaporation rate is the dominant predictor (b* = +0.43 mm/yr) for NW rainfall anomalies, while Niño3.4 SST is the leading predictor (b* = − 0.51 mm/yr) for NE rainfall anomalies, which are both significant at 95% confidence level. These results confirm that the multiyear wet/dry conditions in the NE are more strongly explained by remote multiyear variations of ENSO-like tropical Pacific SST anomalies, while the multiyear rainfall variations for the NW are more strongly influenced by the local rainfall-wind-evaporation feedback. However, as envisioned by Sekizawa *et al*.^8^, this rainfall-wind-evaporation feedback operates only during the monsoon portion of the wet season (January through March) and then fades to zero as the monsoon retreats. Hence, some additional memory would appear to be required to sustain multiyear persistence of NW rainfall anomalies.

**Table 1:** Partial regression coefficients of area-averaged rainfall anomalies with standardized Niño3.4 SST and local evaporation indices for period 1901–2010. Values significant above the 95% confidence level are shown in asterisk.

Indices	Niño3.4	Local Evaporation
Rain NW	−0.21	0.43*
Rain NE	−0.51*	0.22

#### Soil moisture as a possible source of memory

Slow-varying land-surface processes can impact the local atmospheric variability, and soil moisture memory (defined as the persistence of soil moisture) can contribute significantly to such land-climate interactions. Soil moisture-atmosphere feedback has been shown to promote memory to sustain extreme hydrological conditions such as droughts and floods^25^, and improved initialization of soil moisture has been shown to improve seasonal prediction of Australian climate^10,26^. Here, we explore the possible contributions of soil moisture and evapotranspiration feedbacks to multiyear rainfall variations using the latest AWRA-Lv6 analyses^20^ that includes soil moisture and evapotranspiration (across Australia and are available from 1912 onwards (see Methods).

**Figure 6:**
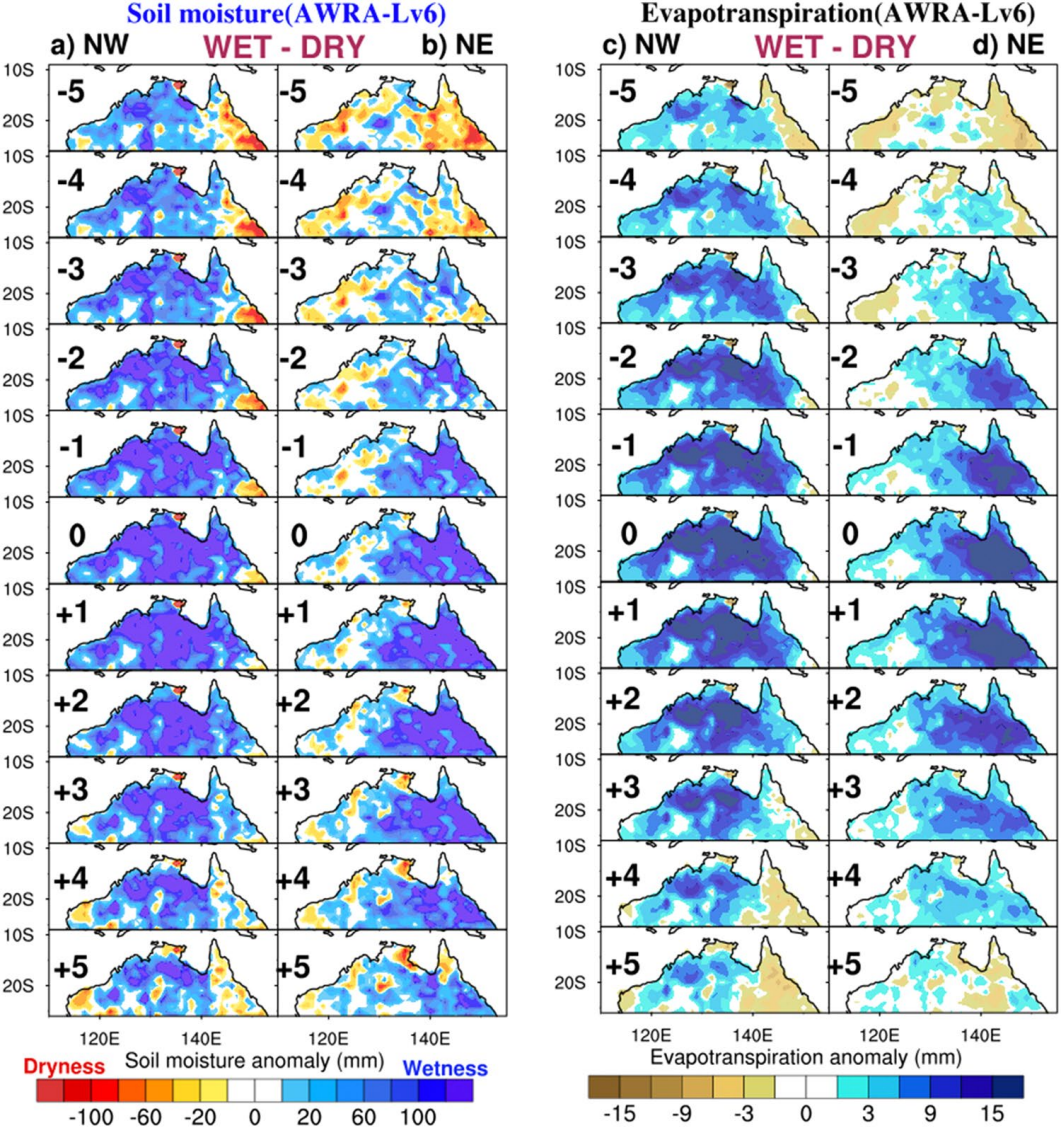
Lagged composite (years −5 to years +5) for WET minus DRY years of vertically-integrated soil moisture (**a**) NW and (**b**) NE and evapotranspiration for (**c**) NW, and (**d**) NE at multiyear timescale. All data have been detrended and low pass filtered with a 5-year running mean.

The temporal evolution of column-integrated soil moisture and evapotranspiration anomalies for the wet-dry composite for the NE and the NW is displayed in Fig. 6. In order to depict the temporal evolution of the anomalies, we make composites beginning 5 years prior and extending to 5 years after the peak occurrence of NE and NW rainfall. As for the previous analyses, we make use of data that are averaged Nov–Apr and low pass filtered with a 5-year running mean. For the NW, precursor enhanced soil moisture (Fig. 6a) and evapotranspiration (Fig. 6c) anomalies are detectable across much of north western Australia up to 5 years in advance of peak NW rainfall (lag 0). In contrast, enhanced soil moisture (Fig. 6b) and evapotranspiration (Fig. 6d) over the north east occur more coincident with or even peaking after enhanced NE rainfall, with little indication of any precursor enhancement. Similar evolution is depicted using evaporation from ERA-20C^19^ (Supplementary Fig. S6).

**Figure 7:**
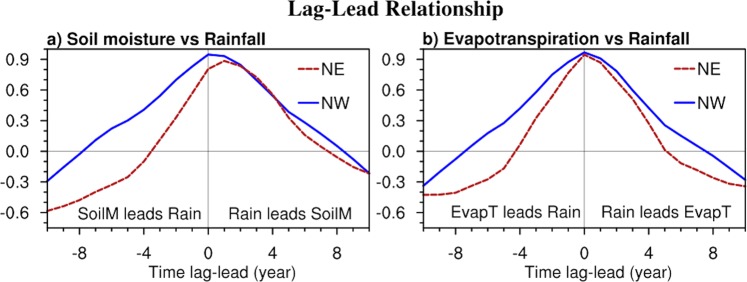
Lag-lead correlation of (**a**) rainfall and soil moisture (SoilM), and (**b**) rainfall and evapotranspiration (EvapT) for NW (blue, solid curve), and NE (red, dashed curve). All data have been detrended and low pass filtered with a 5-yr running mean.

We further quantify this contrasting lead-lag relationship between the NE and NW by calculating the lagged correlation of area mean rainfall with area mean soil moisture (Fig. 7a) and area-mean evapotranspiration (Fig. 7b). Peak NE soil moisture lags by one year and is asymmetric with respect to lag 0 (i.e. the leading relationship drops off more rapidly than the lagging relationship). The associated evapotranspiration shows no lag and drops off quickly with increasing lag. In contrast, NW soil moisture peaks at zero lag, and has a much stronger precursor relationship than NE soil moisture does. Similarly the evapotranspiration shows a much slower drop off both preceding and following enhanced rainfall. The autocorrelations of rainfall, soil moisture and evapotranspiration (not shown) indicate enhanced autocorrelation up to 5 years lead for the NW but the autocorrelation drops off much quicker for the NE. Soil moisture-rainfall feedback thus appears to be a possible mechanism that can enhance multiyear variations of NW rainfall but soil moisture appears to mainly follow NE rainfall with limited evidence of any cooperative feedback.

## Conclusions

**Figure 8:**
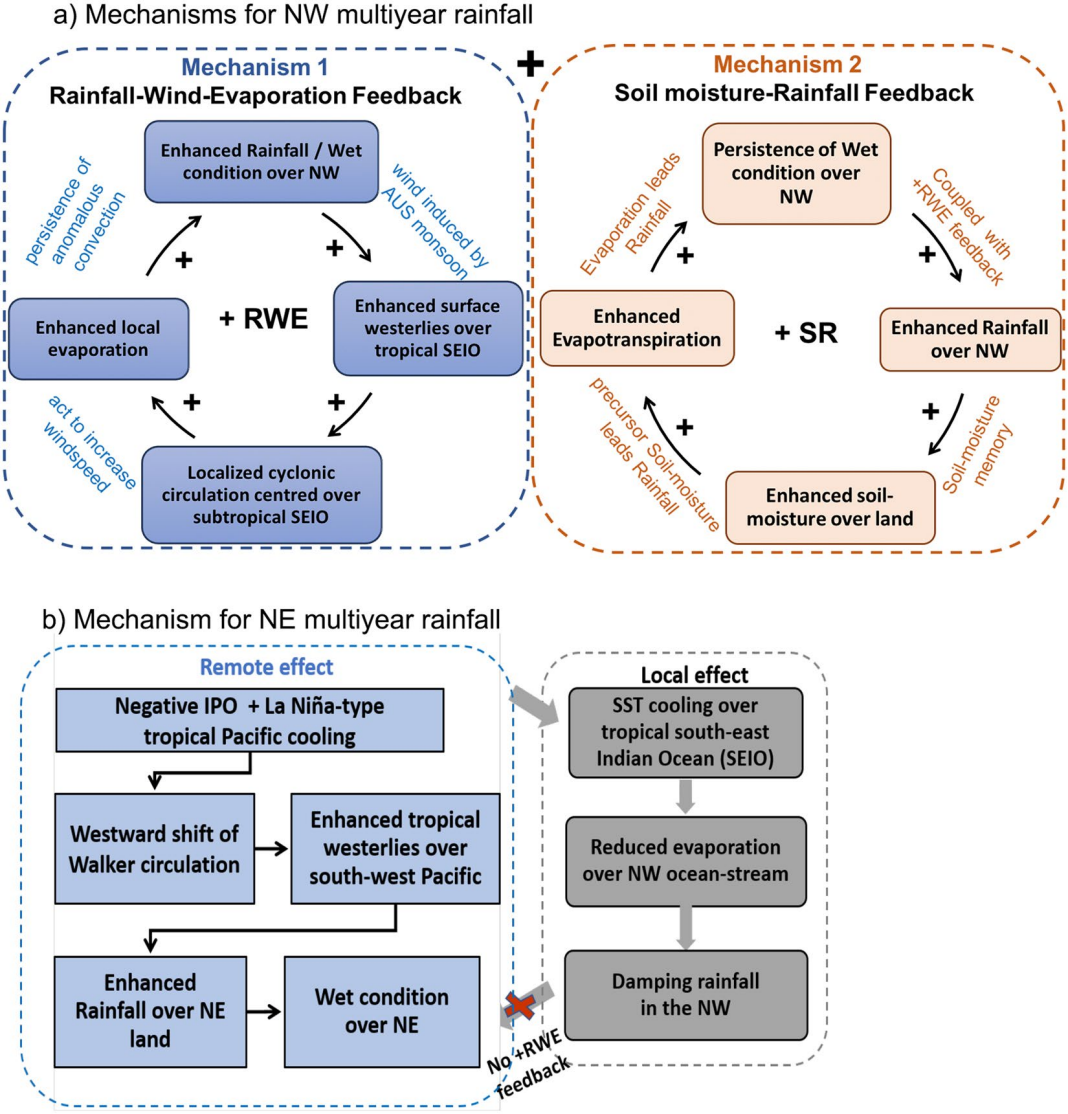
Schematic of the driving mechanisms for multi-year rainfall variability over (**a**) NW, and (**b**) NE Australia.

We have identified a regional asymmetry in multiyear variations of wet-season rainfall across northern Australia and have proposed distinctive driving mechanisms based on analyses for the period 1900–2017. Multiyear rainfall variability in NW Australia is largely independent of remote forcing from low frequency variations of ENSO and the IPO in the tropical Pacific. It appears to be promoted locally by a combination of rainfall-wind-evaporation and soil moisture-rainfall feedbacks, with soil moisture serving as a source of multiyear memory. In contrast, multiyear rainfall variations in NE Australia are largely influenced remotely from the tropical Pacific, with the local feedback to the north west of Australia largely being negative so to damp the remote influence from the Pacific on the NW. This damping in the NW thereby acts to concentrate the ENSO/IPOs promoted variations to the NE. Figure 8 summarises contrasting mechanisms for promoting multiyear wet/dry conditions for NW and NE Australia. Figure 8a demonstrates how the degree of persistence of multi-year wet conditions over the NW Australia is primarily regulated by a combination of local rainfall-wind-evaporation feedback and soil moisture-rainfall feedback. First, enhanced rainfall in the NW will drive enhanced surface westerlies over the tropical south-eastern Indian Ocean to the north west of Australia. These westerly anomalies, which are part of a localized cyclonic circulation anomaly centred over the subtropical eastern Indian Ocean, will act to increase the surface windspeed in conjunction with the seasonal development of monsoon westerlies across northern Australia. These enhanced windspeed will act to increase evaporation over the warm ocean to the north west of Australia and thereby feed enhanced rainfall over the land^8^. This enhanced rainfall promoted by the local rainfall-wind-evaporation feedback will increase soil moisture over the land, which then can provide long-term memory to promote enhanced evapotranspiration in succeeding year. Comparable, but opposite feedbacks can also generate and sustain multiyear dry conditions in the NW.

In contrast, the multiyear rainfall variability across the NE is primarily controlled by the remote influences from ENSO/IPO in the tropical eastern Pacific (Fig. 8b). During the wet years, the La Niña-type tropical Pacific SST anomaly cause a westward shift of the Walker circulation with enhanced upward motion over Australian longitudes and enhanced rainfall in the NE. However, the associated sustained westerly anomalies over the tropical Indian and western Pacific act to cool the ocean surface, thereby acting to reduce local evaporation to the north west of Australia reducing rainfall in the NW. This damping of rainfall in the NW when rainfall is enhanced in the NE also means that soil moisture in the west will provide little long term memory. The contrasting behaviour of soil moisture between NE and NW during wet/dry conditions is an expression of the different processes controlling rainfall in each region.

The implications of this study are that both NW and NE multiyear rainfall variations may be potentially predictable but deriving from different sources. For the NE, predictability would appear to be constrained by the predictability of low frequency variations of ENSO or the IPO. Although to date little predictability of multiyear variations of tropical Pacific SST has been demonstrated^27,28^, there is hope that with improved model and ocean initialization, some predictability of the low frequency ENSO in the Pacific can be achieved^29,30^. In contrast, the predictability of NW multiyear rainfall variations would appear to depend in part on initial soil moisture conditions. However, tapping into this source of predictability may be challenging because atmosphere-land surface interactions are notoriously difficult to faithfully model^25^. Experiments whereby soil moisture is constrained during forecasts have shown a large impact on prediction of seasonal climate^10^, but little attention to date has been given to multiyear timescales. Ongoing predictability experiments are now being conducted to assess this potential.

## Methods

### Precipitation data

Precipitation data were obtained from high-resolution (0.05° × 0.05°) gridded rainfall analyses (1900–present) developed by the Bureau of Meteorology’s Australian Water Availability Project (AWAP^15^). These analyses are based on available station observations across the Australian continent and are available 1900–present. Our analysis is performed on a monthly averaged and interpolated 0.25° × 0.25° grid rainfall data.

### Soil moisture data

We utilize monthly averaged, multi-layer (three layers, integrated from depth of 0 to 5 m) soil moisture and evapotranspiration analyses from the Australian Water Resources Assessment system’s landscape model (AWRA-L version 6^20^). These analyses are available on a 0.05° × 0.05° grid for the period 1912–2017. AWRA-Lv6 is a water balance model that is driven by observed meteorological parameters and has been shown to agree well with independent observations of soil moisture, evapotranspiration and runoff. The data are available on request from the Australian Bureau of Meteorology (http://www.bom.gov.au/water/landscape). Here, we make use of vertically integrated (soil depth 0–5 m) soil moisture and evapotranspiration.

### SST data and ERA20C reanalysis

Monthly mean SST analyses of 1° × 1° grid resolution from the Hadley Centre Global Sea Ice and Sea Surface Temperature (HadISST^18^; https://www.metoffice.gov.uk/hadobs/hadisst/) were analysed for the period 1900–present. The atmospheric variables (surface wind, mean sea level pressure, evaporation rate) on a 1° × 1° grid resolution were obtained from the ECMWF twentieth-century reanalysis (ERA20C^19^, https://rda.ucar.edu/datasets/ds626.0/) which is available for the period 1900–2010.

### Multiyear variability

We first calculate the wet season (November to April) averaged timeseries for each year from 1901–2017, labelling the year based upon January. For instance, year 1980 is the average of November 1979 to April 1980. The multiyear component is defined as the low-pass filtered timeseries by smoothing the interannual timeseries with a 5-year running mean. The results are not sensitive to the cut-off frequency of this low pass filter. All timeseries have been detrended using a least-square fit to better emphasize the multiyear variations before analysis.

### Rotated empirical orthogonal function analysis

The orthogonality constraint imposed by simple EOFs can cause difficulty in interpreting resulting space-time patterns as the physical modes may not be necessarily orthogonal. The advantage of the rotated EOF (rEOF) is that this method can diminish such limitations as the rotation can transform the EOFs into a non-orthogonal linear basis and can better discriminate to localized but possibly more physically meaningful patterns. In this study, we apply rEOF based on the varimax method^14^ to identify homogenous rainfall regions with dominant patterns of multiyear rainfall variability over Australia during northern wet-season (Nov–Apr). We first decompose the multiyear rainfall field into EOFs and PCs and based on a ‘scree’ test criterion, a subset of first 6 EOFs were chosen to be rotated. The largest fraction of variance explained by the rEOF1 is 21.5% which corresponds to the homogenous rainfall pattern over the northeast Australia, while rEOF2 describes 18.5% variance corresponding to a homogenous rainfall over northwest Australia. The estimated rotated PC1 and PC2 are in addition normalized to have unit standard deviation and used to identify two homogenous rainfall regions over northern Australia (Fig. 2).

### Climate indices

We adopted two different SST indices to depict key variability in the tropical Pacific: the Niño3.4 index over the eastern equatorial Pacific (5°S-5°N, 120°-170°W) is the common index used to depict interannual variability associated with ENSO. The tripole index (TPI)^16^ (https://www.esrl.noaa.gov/psd/data/climateindices/list/) is a standard index to capture key variability associated with the IPO. The low-frequency Niño3.4 and TPI indices yield nearly identical information about the state of the multiyear variation of the tropical Pacific. The correlation between Nov–Apr averaged Niño3.4 and TPI indices, after smoothing with a 5-year running mean, is ~0.95. We thus primarily use the low pass filtered Niño34 index to depict the low frequency state of the tropical Pacific.

### Statistical analysis

We use linear correlation analysis to study the teleconnection between global SST and rainfall timeseries. We perform a Fast Fourier Transform analysis using NCL functions (https://www.ncl.ucar.edu/) to calculate periodograms with detrended and tapered time series data. We estimate cross-spectra and the coherence-square statistic to identify significant frequency-domain correlation between the two timeseries after smoothing the periodograms using modified Daniell smoothing. We also compute the phase estimates in the cross-spectrum useful where significant frequency-domain coherence-squared exists. We also apply composite analysis based on the multiyear wet and dry epochs identified from NW and NE rainfall indices. In addition, we apply a multiple linear regression analysis to identify the relative contribution of local and remote forcing for promoting NW and NE rainfall variations, and a lag-lead correlation is also applied to study the feedback between rainfall and soil moisture.

### Significance testing

The statistical significance is tested by a two-tailed Student’s t-test. For multiyear, each non-overlapping 5-year mean was considered independent. We report statistical significance at the 95% confidence level, unless otherwise specified.

### Graphic software

All figures were generated by The NCAR Command Language (Version 6.4.0) [Software]. (2019). Boulder, Colorado: UCAR/NCAR/CISL/TDD. 10.5065/D6WD3XH5.

## Supplementary information

Supplementary Information.

## Data Availability

All data generated and analysed are available from the corresponding author upon request.
