# Supplementary material for: Mechanisms of multiyear variations of Northern Australia wet-season rainfall
Source: Sci Rep. 2020 Mar 20;10:5086. doi: 10.1038/s41598-020-61482-5 (PMC7083836; doi:10.1038/s41598-020-61482-5)
Supplement: Supplementary file 1 — Supplementary Information. [file 41598_2020_61482_MOESM1_ESM.pdf]

Mechanisms of multiyear variations of Northern Australia wet-season rainfall

S. Sharmila[1,2*] and H. H. Hendon[2]
[1] *Centre for Applied Climate Sciences, University of Southern Queensland, Australia*
[2] *Bureau of Meteorology, Melbourne, Australia*
*Email: sharmila.climate@gmail.com/sharmila.sur@bom.gov.au

Supplementary Figures: S1, S2, S3, S4, S5, S6

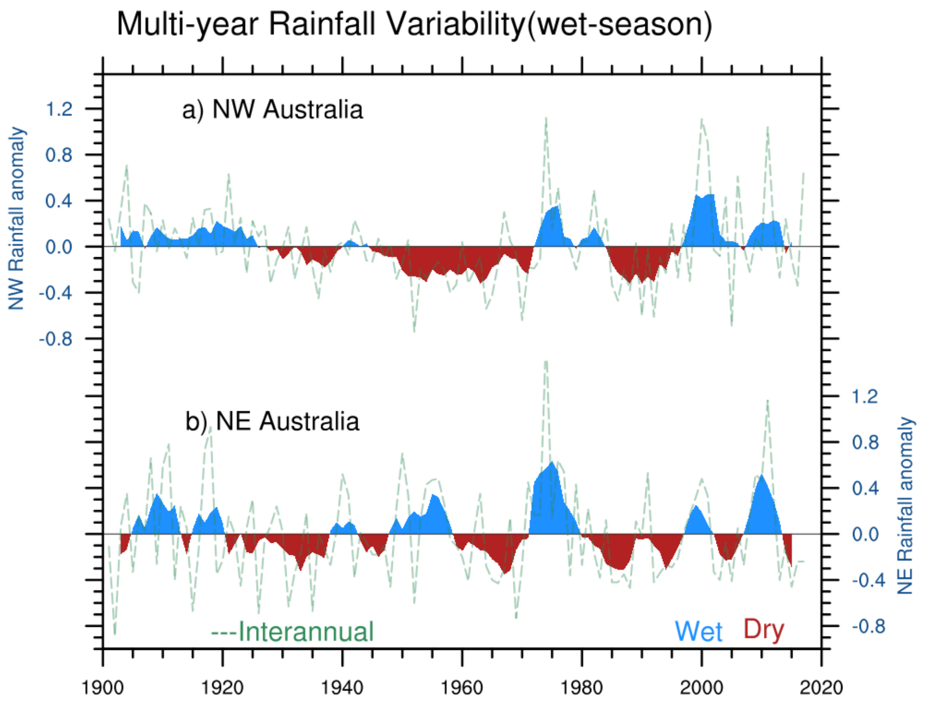

Suppl. Fig S1 | Detrended and low pass filtered (5-year running mean) wet season rainfall anomalies(shading) over NW and NE Australia. The unfiltered wet-season averaged interannual anomalies are plotted by thin green dashed curves.

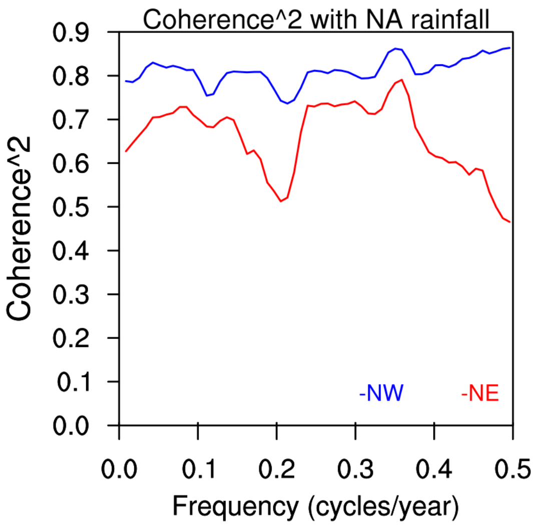

Suppl. Figure S2 | Coherence-squared spectra of area-averaged northern Australia (NA) rainfall with NW (blue), and NE (red) rainfall.

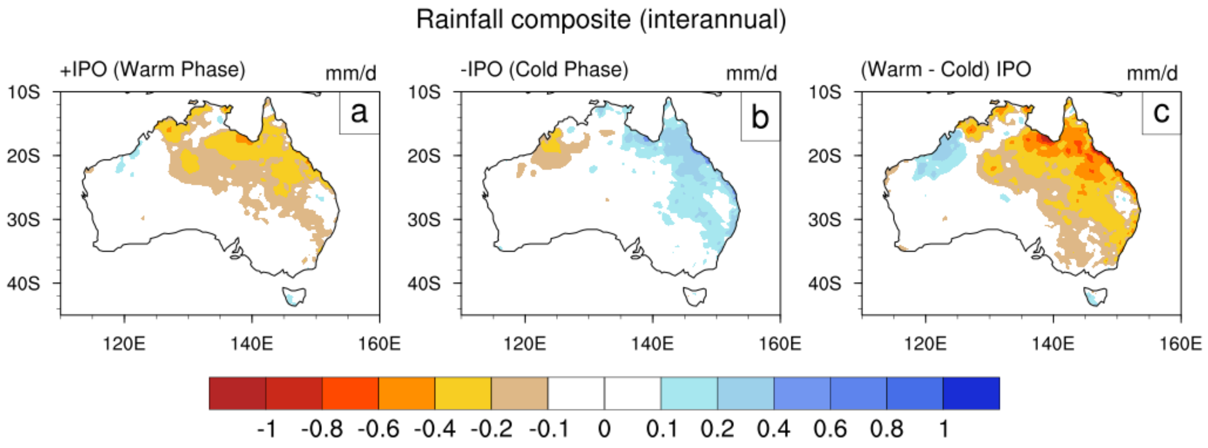

Suppl. Figure S3 | Rainfall composites for a) +IPO warm phase, b) -IPO cold phase and c) their difference (warm - cold) based on AWAP rainfall.

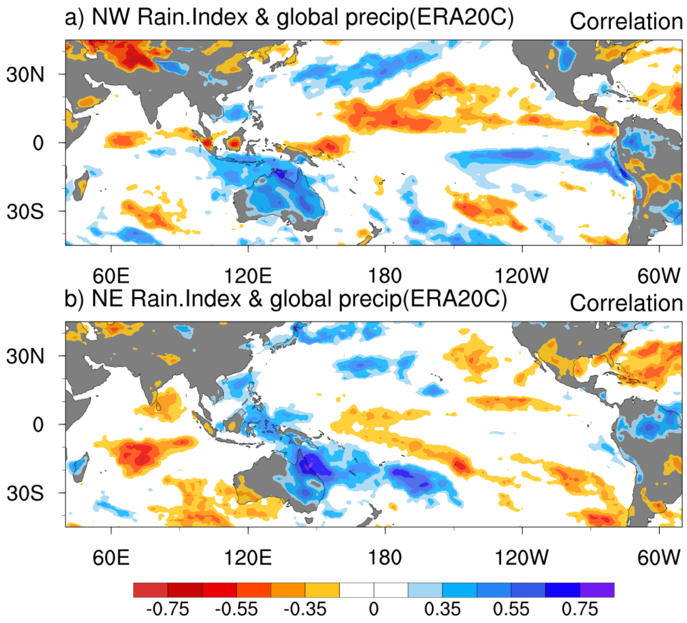

Suppl. Figure S4 | Correlation of ERA-20C global precipitation (shaded, mm/day) with rainfall indices of a) NW, and b) NE respectively.

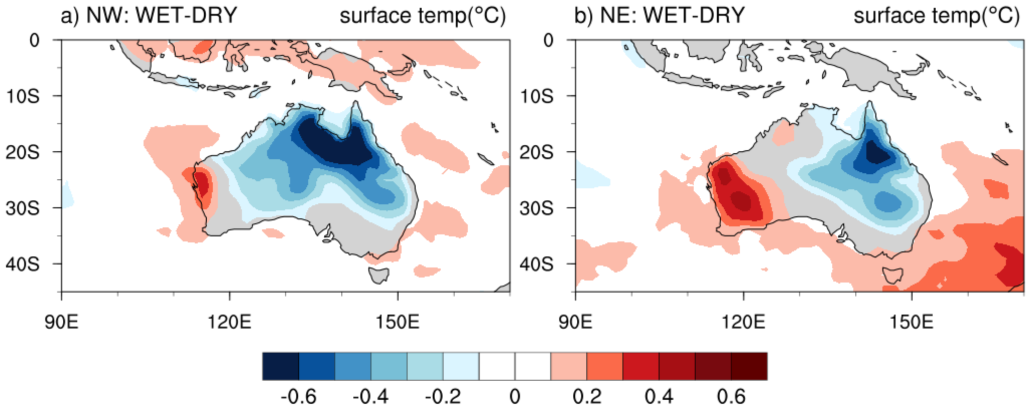

Suppl. Fig. S5 | WET minus DRY composites of surface air temperature for (a) NW and (b) NE based on ERA20C reanalyses. All data have been detrended and low pass filtered with a 5-year running mean.

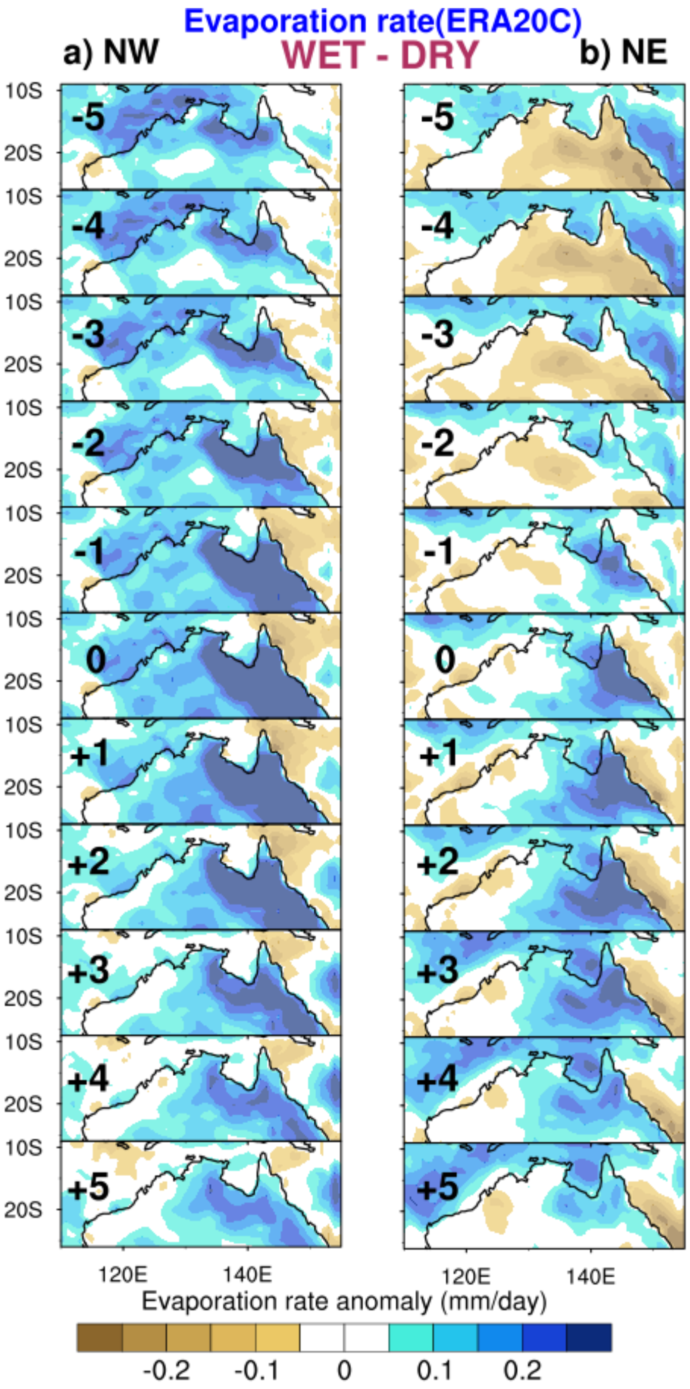

Suppl. Figure S6 | Lagged composite (years -5 to years +5) for WET *minus* DRY years of the evaporation rates (unit mm d^{-1}) based on ERA-20C for the period 1901-2010. All data have been detrended and low pass filtered with a 5-year running mean.
